# Prevalence of gastrointestinal manifestations among diabetic patients in the Aseer region: A cross-sectional study

**DOI:** 10.1097/MD.0000000000039895

**Published:** 2024-09-27

**Authors:** Ayoub Ali Alshaikh, Ahmed Aedh Ahmed Alshehri, Abdullah Zaher Abdullah Alshehri, Abdulmohsen Saleh Alobaid, Ali Alhussein Mohammed, Turki Saeed F Alshahrani, Abdulrhman Zaher Mohammed Albarqi, Hamad Saud Hassan Sultan, Mohammed Alhussen, Ali Dhafer Ali Shehri, Ramy Mohamed Ghazy

**Affiliations:** a Family & Community Medicine Department, College of Medicine, King Khalid University, Abha, Saudia Arabia; b Medical Colleague, College of Medicine, King Khalid University, Abha, Saudia Arabia; c Tropical Health Department, High Institute of Public Health, Alexandria University, Alexandria, Egypt.

**Keywords:** diabetes duration, diabetes mellitus, gastrointestinal symptoms, HbA1C, health insurance

## Abstract

Diabetes mellitus (DM) has a systemic consequence, influencing many systems of the body, including metabolic functions. This study aimed to determine the prevalence of gastrointestinal complications among patients with type 2 DM in the Asser region of Saudi Arabia, identify sources of information, and investigate the association of these symptoms with disease duration and glycated hemoglobin. This cross-sectional study was conducted between November 13 and December 27, 2023. The questionnaire collected demographic data including age, sex, education, employment, income, and nationality, and 16 questions (5 points for each symptom) about the frequency of gastrointestinal symptoms in the past 3 months. The total score was 80, participants were categorized based on their total scores into 2 groups: those scoring 40 or below, and those scoring above 40. A total of 230 patients were included in this study, their median age was 32.0 (24.00) years, 60% were men, 63.9% were married, 38.7% earned between 5000 and 10,000 Saudi Riyal/month, 85.2% did not work in the medical field, 39.1% held university degrees, 54.8% did not have health insurance, 70.4% did not smoke, 35.7% worked in government jobs, 63% lived in urban areas, 95.2% were Saudi and 53.5% had only DM. More than half of the respondents, 57.4%, relied on doctors for information about DM. Dysmotility symptoms were common: dyspepsia affected 26.5% often and 5.7% always; early satiety impacted 24.3% often and 5.2% always; and bloating affected 28.3% often and 10.9% always. Constipation/diarrhea were a common complaint, with 23.5% of patients experiencing them often and an additional 4.8% reporting it always. Stool consistency also varied widely, with 21.7% experiencing lumpy or hardened stool. Health insurance status and having chronic diseases showed significant association with the severity of symptoms. Duration of diabetes and glycated hemoglobin were associated with the frequency of the symptoms. Gastrointestinal symptoms are common among diabetic patients in Aseer. The frequency of symptoms is associated with glycemic control, duration of diabetes, and health insurance status. These findings highlight the need for improved management and support for better gastrointestinal health in diabetes.

## 1. Introduction

One in 10 people worldwide, or 537 million adults aged 20 to 79 years, have diabetes mellitus (DM), a serious health issue. This startling number is anticipated to rise to 643 million by 2030 and a mind-boggling 783 million by 2045. Concerningly, the disproportionate burden of this disease is highlighted by the fact that more than 75% of adults with DM live in low- and middle-income countries. DM has an impact that goes beyond only its prevalence; in 2021, it caused 6.7 million fatalities, or 1 death every 5 seconds.^[1]^ Type 2 diabetes mellitus (DM2) accounts for 90% of all diabetes cases and is distinguished by insufficient insulin secretion by pancreatic islet cells, tissue insulin resistance, and an insufficient compensatory insulin secretory response.^[2,3]^

DM2 and prediabetes (a condition that precedes diabetes) are referred to as dysglycemia or insufficient regulation of blood glucose levels. Those with prediabetes have a 5 to 10 times higher annual risk of getting DM2 than those with normal glucose tolerance and blood glucose levels.^[4]^ The development and progression of DM2 are affected by a variety of risk factors, both modifiable and non-modifiable. Modifiable risk factors include being overweight, sedentary, having poor dietary habits, having hypertension, and smoking. Age, family history, ethnicity, and genetics are examples of unmodifiable factors.^[5]^

DM has grown 3-folds in Saudi Arabia over the last 30 years, making it a serious medical issue due to its high mortality, morbidity, and vascular complications.^[6–8]^ Saudi Arabia has the second-highest prevalence of DM in the Middle East and ranks seventh globally.^[9]^ Nearly 3 million individuals have prediabetes, while it is believed that over 7 million people have DM.^[6]^ In Saudi Arabia, an epidemiological health study was conducted on individuals aged 30 to 70 years who resided in selected households, 4004 of the 16,917 survey respondents, or roughly 23.7% of the population, had been diagnosed with DM.^[10]^ However, other studies showed that Saudis have higher prevalence rates of DM, ranging from 26.0% to 61.8%.^[8,11,12]^

DM has a systemic consequence, influencing many systems of the body, including metabolic functions. The duration and intensity of hyperglycemia have a strong correlation with the degree and severity of organ involvement, with chronic exposure resulting in progressive and multi-organ dysfunction.^[5,13]^ Gastrointestinal symptoms are commonly reported in DM compared to nondiabetic^[14]^ and are often attributed to autonomic neuropathy.^[15]^ It has been indicated that inadequate glycemic control, especially when accompanied by neuropathy and nephropathy, can alter individuals’ perception of gastrointestinal pain.^[16]^ In general, gastrointestinal symptoms occur in 30% to 76% of patients^[16–18]^ with more prevalence among women.^[19]^ The prevalence of these symptoms varies by ethnic group.^[16]^ So, improving diabetic care and the affected patient’s quality of life requires early detection and effective management of gastrointestinal problems.^[20]^

This study hypothesized that there is a high prevalence of gastrointestinal symptoms among diabetic patients. The primary objective of the study was to evaluate the prevalence of gastrointestinal complications among diabetic patients in the Aseer region and explore the association between these symptoms and factors such as the duration of diabetes and glycemic control. Additionally, the study aimed to identify sources of health information about DM, providing insight into how patients acquire knowledge about their medical condition and its management.

## 2. Subjects and methods

### 2.1. Study design

This cross-sectional study was conducted between November 13 and December 27, 2023, to assess the prevalence of gastrointestinal symptomps among diabetic patients in the Aseer Region, Saudi Arabia. Data was collected using a face-to-face interview.

### 2.2. Sample size and study population

The sample size was calculated using General Power Analysis Software (G*Power Epi-Info) software with a margin of error of 5% and a power of 80%. Assuming that 57.8% of participants had gastrointestinal symptoms associated with DM, a sample size of 189 participants was determined. We increased the sample to 230 to compensate for a nonresponse rate of 15%.

Inclusion criteria: The study included diabetes individuals (diagnosed for at least 1 year) from the Aseer region aged 18 to 80 years who had given informed consent. Participants must have had a confirmed diagnosis of DM.

Exclusion criteria: We excluded people who had gastrointestinal symptoms from nondiabetic causes, such as peptic ulcer disease or inflammatory bowel disease. Patients with significant comorbidities that could influence the study’s findings, such as advanced renal failure or severe hepatic illness, were also excluded. Pregnant patients were excluded because pregnancy may have independent impacts on gastrointestinal symptoms. Individuals who had recently undergone major gastrointestinal surgery, as well as those with considerable cognitive impairment, were excluded from the study. Finally, patients taking drugs that could severely impair gastrointestinal symptoms or glycemic management, such as high-dose steroids, were excluded from the trial.

### 2.3. Sampling technique

A non-probability convenience sampling technique was employed to select participants based on the inclusion and exclusion criteria. The questionnaire was collected from diabetic patients attending outpatient clinics at King Khalid University, Aseer region.

### 2.4. Data collection

#### 2.4.1. The questionnaire consists of 2 sections

Demographic data was collected in the first section: The demographic data of the respondents, such as their age, sex, marital status, level of education, employment, monthly income in Saudi Riyal (1 United States Dollar = 3.75 Riyal), and nationality were the focus of this section.

The second section consists of 16 questions about how frequently gastrointestinal symptoms that were troublesome in the previous 3 months had occurred. On a 5-point Likert scale, the frequency of each symptom was scored and recorded as not at all, rarely, occasionally, often, or very often. The total score for the 16 questions was 80. Participants were categorized based on their total scores into 2 groups: those scoring 40 or below, and those scoring above 40. When the symptom was reported to occur frequently or very frequently, a positive response was recorded for this analysis. All symptoms that did not fully explain themselves were accompanied by the following standard description: Dysphagia (difficulty swallowing, in which solid food or liquids stick on the way down), early satiety (feeling full soon after starting to eat, preventing the person from finishing a normal meal), postprandial fullness (an unpleasant feeling of food remaining in the stomach after a normal meal), bloating (a feeling as though the stomach or abdomen were swollen), heartburn (burning pain or discomfort behind the breast bone rising toward the throat), and urgency (a need to have a bowel movement that made the person rush to the toilet). The first category, esophageal symptoms, included heartburn and dysphagia. The second, upper dysmotility symptoms, encompassed early satiety, postprandial fullness, bloating, nausea, and vomiting. The third category, bowel symptoms, covered a range of issues such as self-reported diarrhea or constipation, loose or watery stools, more than 3 bowel movements per day, urgency, fecal incontinence, fewer than 3 bowel movements per week, lumpy or hard stools, and anal blockage. The fourth category, diarrhea symptoms, included more than 3 bowel movements per day, loose or watery stools, and urgency. Lastly, the fifth category, constipation symptoms, consisted of fewer than 3 bowel movements per week, lumpy or hard stools, and anal blockage.^[15]^

### 2.5. Data management and analysis

Data were statistically analyzed using the Statistical Package for Social Sciences (SPSS) version 27. Categorical variables were summarized using frequencies and percentages, while continuous variables were described using median and interquartile range. The chi-square test was applied to assess associations between demographic factors and the occurrence of gastrointestinal symptoms, with the assumption that the expected frequency in each cell of the contingency table was at least 5. A *P*-value of < .05 was considered the threshold for statistical significance.

### 2.6. Ethical approval

Ethical approval was obtained from the Ethics Committee of King Khalid University (ECM#2023-3106). Each participant digitally signed an informed consent form before participating in the survey.

## 3. Results

Age distribution shows that 31.3% of respondents are between 18 and 34 years, the median age was 32.0 (24.00) years, 60% were male, 63.9% were married, 38.7% earned between 5000 and 10,000 Saudi Riyal, 85.2%, did not work in the medical field, 39.1% held university degrees, 55.2% were covered with health insurance, 70.4% did not smoke, 35.7% worked in government jobs, 63%, lived in urban areas, 95.2% were Saudi, and 53.5% had only DM2 (Table 1).

**Table 1:** Demographic and socioeconomic profile of survey respondents.

Variable	Level	Frequency	Percent
Age category	18–34 years	72	31.3
	35–49 years	63	27.4
	50–64 years	71	30.9
	65–80 years	24	10.4
Gender	Female	92	40.0
	Male	138	60.0
Marital status	Divorced	15	6.5
	Married	147	63.9
	Single	54	23.5
	Widow	14	6.1
Monthly income	<5000 Riyal	76	33.0
	5000–10,000 Riyal	89	38.7
	15,000–20,000 Riyal	38	16.5
	>20,000 Riyal	15	6.5
Work medical field	No	196	85.2
	Yes	34	14.8
Education	Illiterate	3	1.3
	Read and write	9	3.9
	Primary	15	6.5
	Middle	23	10.0
	Secondary	73	31.7
	University	90	39.1
	Postgraduate	17	7.4
Health insurance	Governmental health insurance	75	32.6
	Private health insurance	29	12.6
	Without health insurance	126	54.8
Smoking	No smoking	162	70.4
	Smoker	51	22.2
	Stopped <6 months	17	7.4
Occupation	Governmental work	82	35.7
	No work	69	30.0
	Private work	28	12.2
	Retired	51	22.2
Living	City	145	63.0
	Village	85	37.0
Nationality	Non-Saudi	11	4.8
	Saudi	219	95.2
Chronic diseases	Allergic diseases	12	5.2
	Heart diseases	17	7.4
	Hypothyroidism	16	7.0
	Just diabetes	123	53.5
	Hypertension	52	22.6
	Others	10	4.3

**Figure 1.**
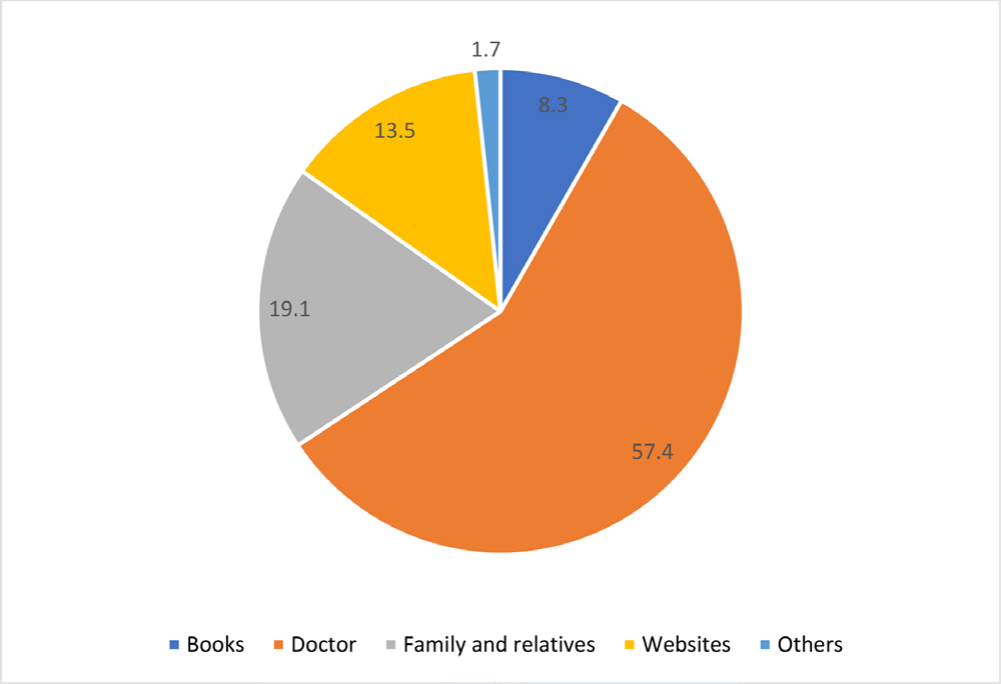
Primary sources of information on diabetes among respondents.

Figure 1 illustrates the primary sources of information on DM among respondents. More than half of the respondents, 57.4%, relied on doctors for information about diabetes. Family and relatives were the next source, accounting for 19.1% of the responses. Websites were also a notable source, used by 13.5% of respondents. Books were the primary source for 8.3% of respondents, while a small percentage, 1.7%, got their information from other unspecified sources.

### 3.1. Esophageal symptoms

**Figure 2.**
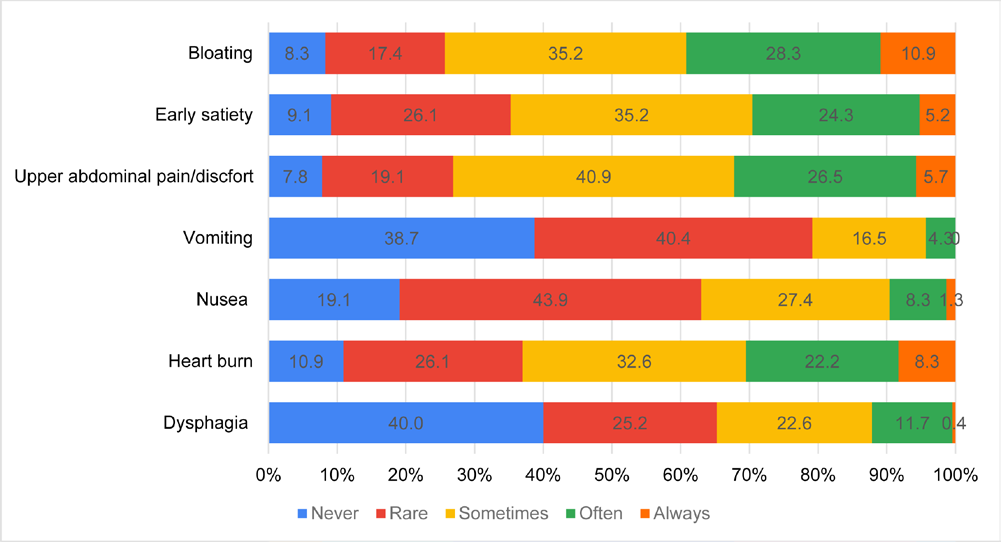
Frequency of upper gastrointestinal symptoms among diabetic patients.

Dysphagia affected 11.7% of patients often and 0.4% always. Heartburn also affected many patients, with 22.2% experiencing it often and 8.3% always (Fig. 2).

### 3.2. Dysmotility symptoms

Dyspepsia emerged as a commonly encountered symptom, with 26.5% of patients experiencing it often and 5.7% reporting it always. Similarly, early satiety was prevalent, with 24.3% of patients experiencing it often and 5.2% always, with 28.3% of patients experiencing it often and 10.9% reporting it always. Nausea, while present, was less frequently reported compared to other symptoms, with 8.3% of patients experiencing it often and only 1.3% always. Vomiting was relatively rare, with 4.3% of patients experiencing it often and none reporting it always (Fig. 2).

### 3.3. Bowel, diarrhea, and constipation symptoms

**Figure 3.**
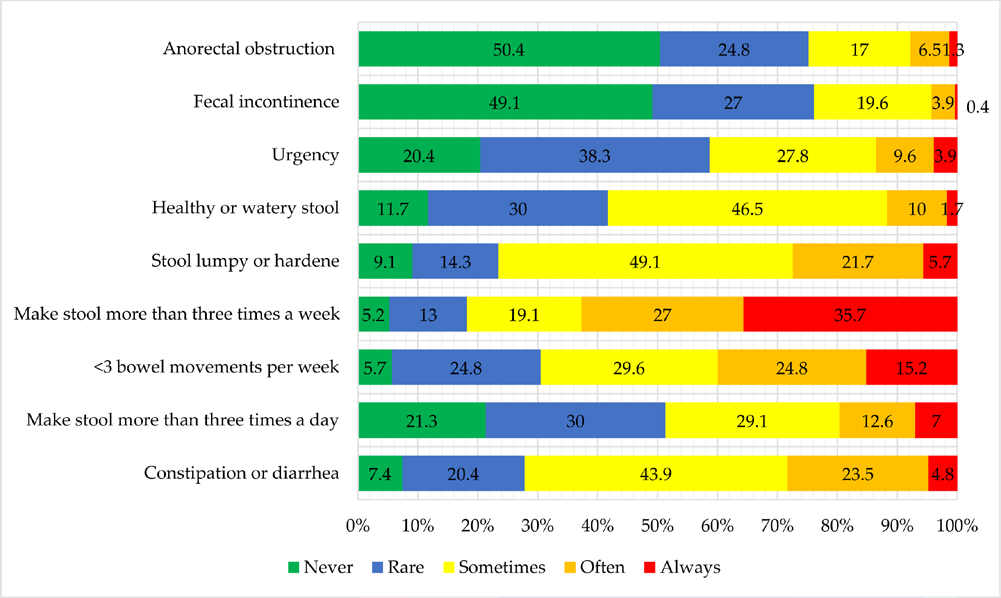
Prevalence and Frequency of lower gastrointestinal symptoms among diabetic patients.

Constipation/diarrhea emerged as a prevalent issue, with 23.5% of patients experiencing them often and an additional 4.8% reporting it always. Similarly, both frequent (more than 3 times a day) and infrequent (less than 3 times a day) bowel movements were reported frequently, with significant percentages experiencing these symptoms often and always. Stool consistency also varied widely, with 21.7% experiencing lumpy or hardened stool often and 5.7% always, contrasting with 10% often and 1.7% always experiencing loose or watery stool. Urgency in bowel movements affected 9.6% often and 3.9% always. Fecal incontinence, though less common, was distressing for those affected, with 3.9% experiencing it often and 0.4% always. Anorectal obstruction, while less frequent, impacted 6.5% often and 1.3% always (Fig. 3).

Table 2 presents a comparison of various demographic and health-related variables between participants who scored 40 or below and those who scored above 40 on a symptom frequency scale. Age-wise, the proportion of participants scoring above 40 increases with age, particularly notable in the 65 and above group, where 70.8% scored above 40. Gender-wise, both females (64.1%) and males (60.1%) showed a higher proportion of scores above 40, with no statistically significant difference between them (*P* = .542). Marital status showed minimal difference between married and unmarried participants, with similar proportions scoring above 40 (61.9% vs 61.4%, *P* = .945).

**Table 2:** Comparison of symptom frequency scores by demographic and health-related variables.

Variables		Scored ≤ 40		Scored > 40		X^2^	*P*
Age	18–34 years	25	34.7	47	65.3	1.98	.575
	35–49 years	27	42.9	36	57.1		
	50–64 years	29	40.8	42	59.2		
	65 and above	7	29.2	17	70.8		
Gender	Female	33	35.9	59	64.1	0.37	.542
	Male	55	39.9	83	60.1		
Marital status	Not married	32	38.6	51	61.4	0.01	.945
	Married	56	38.1	91	61.9		
Monthly income	15,000–20,000 SAR	16	42.1	22	57.9	0.71	.131
	5000–10,000 SAR	30	33.7	59	66.3		
	<5000 SAR	26	34.2	50	65.8		
Smoking	Nonsmoker	74	41.3	105	58.7	3.24	.072
	Smoker	14	27.5	37	72.5	1.31	.253
Work medical field	No	72	36.7	124	63.3		
	Yes	16	47.1	18	52.9	4.62	.564
Education	Illiterate	2	66.7	1	33.3		
	Read and write	2	22.2	7	77.8		
	Primary	6	40.0	9	60.0		
	Middle	8	34.8	15	65.2		
	Secondary	33	45.2	40	54.8		
	University	30	33.3	60	66.7		
	Postgraduate	7	41.2	10	58.8		
Health insurance	Governmental Health insurance	22	29.3	53	80.3	8.89	.011
	Private health insurance	7	24.1	22	75.9		
	Without health insurance	59	46.8	67	53.2		
Occupation	Governmental work	31	37.8	51	62.2	0.29	.962
	Private work	12	42.9	16	57.1		
	Retired	19	37.3	32	62.7		
	Not working	26	37.7	43	62.3		
Living	City	58	40.0	87	60.0	0.50	.478
	Village	30	35.3	55	64.7		
Chronic diseases	Allergic diseases	8	66.7	4	33.3	29.62	<.001
	Heart diseases	2	11.8	15	88.2		
	Hypothyroidism	1	6.3	15	93.8		
	Just diabetes	58	47.2	65	52.8		
	Hypertension	12	23.1	40	76.9		

1 USD = 3.75 SAR (Saudi Riyal).

Regarding monthly income, higher income categories showed varying proportions of scores above 40, but the differences were not statistically significant (*P* = .131). Smokers were more likely to score above 40 (72.5%) compared to nonsmokers (58.7%), although the difference was not statistically significant (*P* = .072). Participants not working in the medical field were more likely to score above 40 (63.3% vs 52.9%), but this difference was also not significant (*P* = .564). Educational levels showed variations, with the highest proportion of scores above 40 observed in the “Read and Write” category (77.8%). However, small sample sizes in some categories preclude definitive statistical significance. Health insurance status showed significant differences, with participants having governmental (70.3%) or private health insurance (75.9%) more likely to score above 40 compared to those without health insurance (53.2%), and this difference was statistically significant (*P* = .026). Occupational categories revealed no significant differences in scores, with similar proportions scoring above 40 across different groups (*P* = .962). Living environment-wise, participants in villages (64.7%) were slightly more likely to score above 40 compared to those in cities (60.0%), but the difference was not significant (*P* = .478). Chronic diseases showed significant differences (*P* < .001), with participants having heart diseases (88.2%) and hypothyroidism (93.8%) being more likely to score above 40. In contrast, those with allergic diseases and other chronic conditions were more likely to score 40 or below.

Dyspepsia showed a strong positive correlation with HbA1c levels (*r* = .252, *P* = .0001). Similarly, early satiety (*R* = 0.191, *P* = .004) and bloating (*R* = 0.219, *P* = .001) were significantly positively correlated with HbA1c levels. Heartburn also exhibits a significant positive correlation with HbA1c levels (*R* = 0.281, *P* = .0001). Nausea, although less strong, demonstrated a significant positive correlation with HbA1c levels (*R* = 0.155, *P* = .019). In contrast, vomiting (*R* = 0.03, *P* = .653) and dysphagia (*R* = 0.076, *P* = .252) showed no significant correlation with HbA1c levels. Constipation/diarrhea were significantly positively correlated with HbA1c levels (*r* = .136, *P* = .039). Infrequent bowel movements (less than 3 times a day) also showed a significant positive correlation (*r* = .143, *P* = .030). Urgency (*r* = −0.06, *P* = .369) and fecal incontinence (*r* = −0.001, *P* = .986) exhibited no significant correlation with HbA1c levels (Table 3).

**Table 3:** Correlation between gastrointestinal symptoms and HbA1c levels among diabetic patients.

		Hba1c
Dyspepsia	*r*	0.252
	*P*	<.001
Early satiety	*r*	0.191
	*P*	.004
Bloating	*r*	0.219
	*P*	<.001
Heartburn	*r*	0.281
	*P*	<.001
Nausea	*r*	0.155
	*P*	.019
Vomiting	*r*	0.030
	*P*	.653
Dysphagia	*r*	0.076
	*P*	.252
Constipation/diarrhea	*r*	0.136
	*P*	.039
Make stool more than 3 times a day	*r*	0.051
	*P*	.446
Make stool less than 3 times a day	*r*	0.143
	*P*	.030
Urgency	*r*	−0.061
	*P*	.369
Fecal incontinence	*r*	−0.001
	*P*	.986
Stool lumpy or hardened	*r*	0.051
	*P*	.440
Make stool more than 3 times a week	*r*	0.101
	*P*	.126
Loose or watery stool	*r*	0.071
	*P*	.281
Anorectal obstruction	*r*	0.028
	*P*	.671

Early satiety showed a significant positive correlation with the duration of diabetes (*r* = .146, *P* = .027), suggesting that patients who have had diabetes for a longer period were more likely to experience early satiety. Bloating also exhibited a significant positive correlation with the duration of diabetes (*R* = 0.171, *P* = .009). Similarly, heartburn show a significant positive correlation with the duration of diabetes (*R* = 0.159, *P* = .016). In contrast, dyspepsia (*R* = 0.085, *P* = .201) and nausea (*R* = 0.118, *P* = .074) showed no significant correlation with the duration of diabetes. Vomiting (*R* = 0.003, *P* = .961) and dysphagia (*r* = −0.024, *P* = .719) also exhibited no significant correlation with the duration of diabetes. Constipation, diarrhea (*R* = 0.031, *P* = .644), and making stool more than 3 times a day (*r* = -0.09, *P* = .174) showed no significant correlation. Other symptoms, such as making stool less than 3 times a day (*R* = 0.035, *P* = .597), urgency (*R* = 0.022, *P* = .737), fecal incontinence (*R* = 0.007, *P* = .912), stool consistency for lumpy or hardened stool (*r *= −0.066, *P* = .317); for loose or watery stool (*R* = 0.035, *P* = .596), frequency of bowel movements for more than 3 times a week (*r* = −0.006, *P* = .923), and anorectal obstruction (*R* = 0.03, *P* = .647) also showed no significant correlation with the duration of diabetes (Table 4).

**Table 4:** Correlation between frequency gastrointestinal symptoms and duration of diabetes among diabetic patients.

		Duration of diabetes
Dyspepsia	*r*	0.085
	*P*	.201
Early satiety	*r*	0.146
	*P*	.027
Bloating	*r*	0.171
	*P*	.009
Heart burn	*r*	0.159
	*P*	.016
Nausea	*r*	0.118
	*P*	.074
Vomiting	*r*	0.003
	*P*	.961
Dysphagia	*r*	−0.024
	*P*	.719
Constipation or diarrhea	*r*	0.031
	*P*	.644
Make stool more than 3 times a day	*r*	−0.09
	*P*	.174
Make stool less than 3 times a day	*r*	0.035
	*P*	.597
Urgency	*r*	0.022
	*P*	.737
Fecal incontinence	*r*	0.007
	*P*	.912
Stool lumpy or hardened	*r*	−0.066
	*P*	.317
Make stool more than 3 times a week	*r*	−0.006
	*P*	.923
Loose or watery stool	*r*	0.035
	*P*	.596
Anorectal obstruction	*r*	0.03
	*P*	.647

## 4. Discussion

This study aimed to assess the prevalence of gastrointestinal symptoms among patients with DM in the Asser region, Saudi Arabia.

### 4.1. The study’s main findings

Gastrointestinal symptoms are prevalent among diabetic patients, with dyspepsia early satiety, bloating, and heartburn being the most common. Symptoms like nausea, vomiting, and dysphagia are less frequent. The study found significant positive correlations between higher HbA1c levels and symptoms like dyspepsia, early satiety, bloating, heartburn, nausea, and constipation/diarrhea; while vomiting and dysphagia show no such correlation. Additionally, the duration of diabetes was positively correlated with early satiety, bloating, and heartburn, but not with dyspepsia, nausea, vomiting, dysphagia, and various bowel movement characteristics. Furthermore, the study highlighted that doctors are the primary source of diabetes information for respondents.

### 4.2. Interpretation of the main study findings

#### 4.2.1. Prevalence of gastrointestinal symptoms

In the current study, there was a high prevalence of gastrointestinal symptoms among diabetic patients. All studied participants had at least 1 symptom whatever its frequency. This aligns with several studies that reported a high prevalence of gastrointestinal symptoms among patients with diabetes.^[14,21,22]^ Hyperglycemia-associated neuropathy has long been recognized as a major mechanism underlying the pathogenesis of gastrointestinal symptoms, likely mediated through oxidative stress and inflammation, similar to other microangiopathies. This neuropathy can disrupt the normal functioning of the gastrointestinal tract. Additionally, alterations in the levels of enteral hormones, such as incretin-related peptides, including glucagon-like peptide-1, glucagon-like peptide-2, and pancreatic polypeptide, may also play a significant role in the development of these symptoms.^[23]^ This high prevalence of symptoms urges the need to manage digestive issues in diabetic patients through a holistic strategy. This includes dietary changes such as increasing or decreasing fiber intake, using medications like laxatives or antidiarrheals, maintaining stable blood glucose levels through proper treatment adherence, practicing stress reduction techniques, engaging in regular physical activity, ensuring adequate hydration, taking probiotics for gut health, and addressing underlying conditions.^[24]^ Consulting with healthcare professionals is vital in building a customized management strategy to effectively alleviate these symptoms and promote overall well-being in patients with diabetes.^[25]^ Specific lifestyle adjustments that can improve bowel function in patients with diabetes and enteropathy symptoms include progressively increasing fiber intake, remaining appropriately hydrated, and having smaller, frequent meals.^[26]^

In the current study, there was no gender difference associated with the severity of gastrointestinal symptoms. On the other hand, Bytzer et al,^[15]^ reported that in both type 1 and DM2, gastrointestinal symptoms are more common in women, who also experience higher levels of psychosocial distress compared to men.

In the current study, there was a high prevalence of gastrointestinal symptoms if there were other comorbid conditions like heart diseases and hypothyroidism. Health insurance was associated with the frequency of symptoms as well. Indeed, thyroid dysfunction can manifest as gastrointestinal symptoms or exacerbate existing gastrointestinal conditions. The relationship between the thyroid gland and the gastrointestinal tract is bidirectional. Hypothyroidism can influence various gastrointestinal organs and functions, highlighting the interconnectedness of these systems.^[27]^

#### 4.2.2. Source of health information for diabetic patients

DM is a complex, chronic condition that necessitates high-quality clinical care and effective self-management to mitigate its severe health and economic impacts.^[28]^ The source from which information about the disease influences both preventive and therapeutic behaviors. The information source can motivate and sustain the interest of patients with diabetes, encouraging them to actively participate in their treatment, thereby achieving satisfactory results.^[29]^ “Information behavior” is the currently preferred term used to describe the various ways in which humans interact with information, particularly how people seek and utilize information.^[30]^ In this study the main source of information about health conditions is physicians. Similarly, a study conducted in Puru reported that healthcare professionals were the main source of information.^[31]^ This underscores the continued importance of traditional sources of health information in Saudi Arabia, even amidst the widespread use of social media and digital health platforms.^[32]^

#### 4.2.3. Association between glycemic control and gastrointestinal symptoms

In the current study, we found a significant association between glycemic control and gastrointestinal symptoms. A similar finding was reported in previous studies.^[33,34]^ On the contrary, Khalaf et al^[35]^ found that the duration of diabetes did not significantly affect the occurrence of these symptoms. It is worth noting that gastric emptying and postprandial blood glucose levels are closely correlated, with alterations in gastric emptying significantly affecting glucose regulation in both healthy individuals and those with DM2. In diabetes, disruptions in gastric emptying, such as delayed or accelerated emptying, are common. Delayed gastric emptying, known as diabetic gastroparesis, can cause upper gastrointestinal symptoms.^[36]^ In insulin-treated patients, disordered gastric emptying can lead to suboptimal glycemic control. Conversely, interventions that slow gastric emptying, like certain medications, can reduce postprandial blood glucose in DM2. These findings indicate that regulating gastric emptying may be critical for improving glycemic control and relieving gastrointestinal symptoms in diabetic patients. As a result, healthcare practitioners should consider evaluating and treating stomach emptying problemes as part of comprehensive diabetes management.

#### 4.2.4. Duration of diabetes and frequency of symptoms

The association between the duration of diabetes and the frequency of gastrointestinal symptoms is complex. In this study, a longer duration of the disease was associated with a higher incidence of gastrointestinal symptoms including early satiety, bloating, and heartburn. In the same vein, several studies have reported similar findings, indicating that the duration of diabetes may significantly influence the occurrence of these gastrointestinal symptoms.^[37,38]^ On the other hand, Fujishiro^[39]^ revealed that total symptom scores did not increase linearly with disease duration. Moreover, they found that the prevalence of symptoms varied across different gastrointestinal regions over time. Lower abdominal symptoms were particularly prominent both early in the disease course when diabetic complications were still mild, and again in the late stages of the disease. These conflicting findings suggest that, while longer disease duration may contribute to an increase in the prevalence of gastrointestinal symptoms, the link is not straightforward and may be impacted by other variables. More studies are needed to clarify these connections and better understand the factors that influence symptom prevalence across time.

### 4.3. Implications of this study

This study emphasizes the important role of healthcare practitioners in teaching diabetic patients about gastrointestinal issues, given that most patients rely on doctors for information. The high prevalence of symptoms such as dyspepsia, early satiety, bloating, and heartburn among diabetic patients demonstrates the necessity for regular screening and focused therapeutic measures to improve patient quality of life. The positive association between higher HbA1c levels and these symptoms suggests that better glycemic control could minimize gastrointestinal disorders. Furthermore, the study emphasizes the value of accessible and inclusive patient education, utilizing several channels such as family, online sources, and community outreach to ensure that all diabetic patients receive comprehensive care and information on how to manage their gastrointestinal symptoms.

### 4.4. Strengths and limitations

The study has a few limitations. First, it is a cross-sectional study, it only collected data at one moment in time, making it difficult to determine causality between DM and gastrointestinal symptoms. Second, the non-probability sampling technique could influence the generalizability of the findings to the larger diabetes community in the Aseer region. Furthermore, self-reported data on gastrointestinal symptoms may be susceptible to recall bias, since individuals may not accurately remember or report their symptoms. Finally, the study’s use of a Likert scale to assess symptom frequency may lead to subjective interpretations of the scale points, resulting in variations in outcomes.

## 5. Conclusions

The study reveals that diabetic patients in the Aseer Region of Saudi Arabia mostly rely on doctors for knowledge regarding their illness. Dyspepsia, early satiety, bloating, and heartburn are common gastrointestinal symptoms among these individuals, indicating a major need for regular monitoring and particular therapeutic measures. The significant correlation between higher HbA1c levels and the incidence of these symptoms underscores the need for efficient glycemic control in preventing gastrointestinal problems. The findings advocate for enhanced patient education efforts, improved symptom management protocols, and inclusive communication strategies to better support the diabetic population in the region. To address the findings of this study it is recommended to improve healthcare provider training on diabetes-related gastrointestinal complications, implement patient education programs, and establish screening protocols for symptoms. The study emphasizes the importance of maintaining optimal HbA1c levels, inclusive communication strategies, and individualized management plans. Further research is needed to understand the long-term effects of diabetes on gastrointestinal health and identify effective interventions.

## Acknowledgments

The authors extend their appreciation to the Deanship of Research and Graduate Studies at King Khalid University, KSA, for funding this work through the Small Research Group under grant number (RGP1/ 180/45-1446).

## Author contributions

**Conceptualization:** Ramy Mohamed Ghazy.

**Data curation:** Ahmed Aedh Ahmed Alshehri.

**Formal analysis:** Ayoub Ali Alshaikh, Ramy Mohamed Ghazy.

**Methodology:** Abdullah Zaher Abdullah Alshehri.

**Project administration:** Hamad Saud Hassan Sultan.

**Resources:** Abdulrhman Zaher Mohammed Albarqi.

**Software:** Mohammed Alhussen.

**Supervision:** Abdulmohsen Saleh Alobaid.

**Writing – original draft:** Ali Alhussein Mohammed, Ramy Mohamed Ghazy.

**Writing – review & editing:** Turki Saeed F Alshahrani, Ali Dhafer Ali Shehri, Ramy Mohamed Ghazy.
